# *Sophora Tomentosa* Extract Prevents MPTP-Induced Parkinsonism in C57BL/6 Mice Via the Inhibition of GSK-3β Phosphorylation and Oxidative Stress

**DOI:** 10.3390/nu11020252

**Published:** 2019-01-23

**Authors:** Hung-Chi Chang, Keng-Fan Liu, Chia-Jen Teng, Shu-Chen Lai, Shu-Er Yang, Hui Ching, Chi-Rei Wu

**Affiliations:** 1 Department of Golden-Ager Industry Management, College of Management, Chaoyang University of Technology, Taichung 41394, Taiwan; changhungchi@cyut.edu.tw; 2 The Department of Chinese Pharmaceutical Sciences and Chinese Medicine Resources, China Medical University, Taichung 40402, Taiwan; cell77821@yahoo.com.tw (K.-F.L.); l56i56l56y56@yahoo.com.tw (C.-J.T.); 3 Department of Pharmacy, Tung’s Taichung MetroHarbor Hospital, Taichung 43550, Taiwan; grace8306036@yahoo.com.tw; 4 Department of Beauty Science and Graduate, Institute of Beauty Science Technology, Chienkuo Technology University, Changhua City 500, Taiwan; jessica@ctu.edu.tw; 5 Department of Pharmacy, Taichung Hospital, Ministry of Health and Welfare, Taichung, 40343, Taiwan; taic73047@gmail.com

**Keywords:** *Sophora tomentosa*, protocatechuic acid, epicatechin, MPTP, Parkinson’s disease, oxidative stress, free radical scavenging capacity

## Abstract

Sophora species are used as dietary medicines in aging-associated symptoms. *Sophora tomentosa* L. (ST) is a native medicinal plant in Southeast Asia; however, there is no pharmacological literature about ST extract. The present study evaluates the antioxidant phytoconstituent contents and radical scavenging capacities of ST extract. The further investigation was to clarify the neuroprotective mechanism of ST extract against 1-methyl-4-phenyl-1,2,3,6-tetrahydropyridine (MPTP)-induced Parkinsonism by assaying the activities of the dopaminergic system and antioxidant defenses, glycogen synthase kinase 3β (GSK3-β) phosphorylation, and α-synuclein levels in C57BL/6 mice. The results show that ST extract alleviated the motor deficits in MPTP-induced Parkinsonism with four behavioral tests, including a rearing locomotor, catalepsy test, balance beam walking test, and pole test. ST extract reversed the number of tyrosine hydroxylase (TH)-positive neurons in substantia nigra (SN) that had decreased by MPTP. ST extract also restored the decreased levels of dopamine and the expression of tyrosine hydroxylase (TH) in the striatum. Furthermore, ST extract restored the levels of glutathione (GSH) and the activities of antioxidant enzymes, and decreased the elevated levels of malondialdehyde (MDA) in mouse striatum. ST extract also decreased α-synuclein overexpression and GSK-3β phosphorylation in mouse striatum. In vitro, ST extract exerted higher 2,2′-azino-bis(3-ethylbenzothiazoline-6-sulphonic acid) (ABTS) radical scavenging capacities through its higher phenolic contents, especially protocatechuic acid and epicatechin. These results suggest that ST extract has the potential to counteract MPTP-induced motor deficit. The neuroprotective mechanism of ST extract against MPTP-induced Parkinsonism might be related to decreasing GSK-3β phosphorylation and restoring the activities of striatal antioxidant defenses to restore the nigrostriatal dopaminergic function and decrease α-synuclein accumulation.

## 1. Introduction

According to epidemiological statistics, Parkinson disease (PD) is the second most common chronic neurodegenerative disease next to Alzheimer’s disease (AD). The characterized neuropathology of PD patients is the selective progressive degeneration of dopaminergic neurons of the substantia nigra (SN) leading to a depletion of projecting dopaminergic nerve fibers in the striatum. As a result, this neuropathological alteration ultimately causes major clinical symptoms of PD patients, including tremor, rigidity, bradykinesia, and postural instability [1,2,3]. Current evidence supports that many potential factors such as age, genetic aberrations, or environmentally-derived and endogenous neurotoxins cause the occurrence of PD. The environmental exposures or inherited mutation in metabolic pathways might cause the production of toxic substances such as reactive oxygen species (ROS) from endogenous dopamine or environmentally-derived neurotoxins [1,4,5]. The overproduction of ROS leads to oxidative damage, the abnormal processing of cellular proteins such as the accumulation of α-synuclein, and triggers the progressive death of dopaminergic neurons. Some evidences have depicted that the mutation or overexpression of α-synuclein caused the degeneration of dopaminergic neurons [6,7]. The overexpression of α-synuclein in SH-SY5Y neuroblastoma cells led to elevated cellular mortality and synaptic degeneration via the decrease in antioxidant defense and upregulation of glycogen synthase kinase 3β (GSK-3β) [8,9,10]. The overexpression of α-synuclein in transgenic mice was also associated with the elevation of active GSK-3β [10]. Wills et al. have also reported that the accumulation of α-synuclein and the active form of GSK-3β are elevated in the postmortem striatum of PD patients when compared to normal people [11]. Accumulating evidence has revealed that GSK-3β and α-synuclein have a mutual interaction with each other. Moreover, 1-methyl-4-phenylpyridinium (MPP^+^) induced PD features such as the impairment of motor function, α-synuclein accumulation, and neurotoxicity, which could be abrogated by GSK-3β inhibitors [12]. Hence, GSK-3β is a determining factor in the expression and neurotoxicity of α-synuclein. 1-Methyl-4-phenyl-1,2,3,6-tetrahydropyridine (MPTP), a dopaminergic pyridine toxin, was discovered accidentally in the synthesis of meperidine analogues. In the early 1980s, MPTP produced a Parkinsonian syndrome in young abusers after its unintentional self-administration. Most of the biochemical, pathological, and clinical features that were observed in these young addicts correspond to the hallmarks of PD patients. Primates injected with MPTP also produced nearly the same pathological and biochemical changes as observed in PD patients. Following the systemic injection of MPTP, only specific strains of mice—especially C57BL/6 mice—exerted Parkinson’s-like symptoms. MPTP also caused the pathological and systemic symptoms of PD patients involving severe motor deficits such as bradykinesia, rigidity, resting tremors, and postural instability, along with oxidative stress and the accumulation of α-synuclein protein in C57BL/6 mice [3,13,14]. Hence, MPTP is the gold standard toxin-based PD animal model that replicates almost all of the pathological hallmarks of human PD patients. The MPTP-induced PD mouse model is a widely excellent model for assessing the neuroprotective or preservative potential of drugs for the treatment of PD when drugs were administered both before MPTP administration and/or while the toxin is active in the first week post-injection.

Sophora species such as *Sophora flavescens* Ait. (SF, Ku Shen) and *Sophora japonica* L. (SJ, Huaihua) are usually used in the treatment of cardiovascular disorders and aging-associated symptoms in traditional Chinese medicine. Accumulating reports have shown that the above Sophora medicinal plants have antioxidative, anti-inflammatory, and neuroprotective effects [15,16]. SF extract protected against MPP^+^-induced and glutamate-induced neurotoxicology in SH-SY5Y neuroblastoma cells and hippocampal cells [17,18]. SF or SJ extracts protected neuronal damage against focal cerebral ischemia in rats [19,20]. Flavonoids are the major active phytoconstituents regarding the antioxidant and neuroprotective potency [15,18,20]. *Sophora tomentosa* L. (ST) is a local native medicinal plant that has a similar application with these above Sophora species in the tropical coast of Southeast Asia. Early reports indicated that ST extract contained some phenolic and flavonoid compounds and exerted antioxidant activities [21]. However, there is no reported literature about the pharmacological activities of ST. Therefore, the present study evaluates the antioxidant phytoconstituent contents and radical scavenging capacities of ST extract using microtiter spectrophotometric methods and high-performance liquid chromatography with photodiode array detector (HPLC-DAD). Then, we want to evaluate the neuroprotective effects of ST extract against MPTP-induced Parkinsonism in C57BL/6 mice. We further elaborate the mechanism of this neuroprotective effect of ST extract against MPTP-induced Parkinsonism by assaying the activities of the dopaminergic system (the levels of dopamine (DA), its metabolites (3,4-dihydroxyphenylacetic acid (DOPAC) and homovanillic acid (HVA)), and the expression of tyrosine hydroxylase (TH)) and antioxidant defense, GSK3-β phosphorylation, and α-synuclein levels in the mouse striatum.

## 2. Materials and Methods 

### 2.1. Plant Collection and Preparation

*Sophora tomentosa* L. (ST) was collected from the wild in Taichung and identified by Chi-Rei Wu of China Medical University in Taiwan. The dried leaves of ST were crushed and sieved through 80 mesh. The collected powders (1.7 kg) were added with 10× (*w*/*v*) distilled water and boiled for one hour, twice. The resulting extracts were concentrated under reduced pressure to obtain ST extract (70.51 g, 4.15 % yield). For in vitro antioxidant assay and HPLC-DAD assay, the ST extract was dissolved in distilled water. For the neuroprotective evaluation on the MPTP-induced PD mouse model, ST extract was dissolved with 0.5% carboxymethylcellulose (CMC).

### 2.2. Chemicals

1,1-diphenyl-2-picryhydrazyl (DPPH), 1-methyl-4-phenyl-1,2,3,6-tetrahydropyridine (MPTP), 2,2′-azino-bis(3-ethylbenzothiazoline-6-sulphonic acid) (ABTS), 5,5′-dithio-bis-2-nitrobenzoic acid (DTNB), 6-hydroxy-2,5,7,8-tetramethylchroman-2-carboxylic acid (trolox), aluminum nitrate, DOPAC, dopamine, ferrous sulfate heptahydrate, Folic-Ciocalteu’s phenol reagent (FCP), gallic acid, gallocatechin, glutathione (GSH), glutathione peroxidase (GPx), glutathione reductase (GR), HVA, malodialdehyde (MDA), quercetin, sodium molybdate, sodium nitrate, superoxide dismutase (SOD), thiobarbituric acid (TBA), trichloroacetic acid (TCA), verbascoside, xanthine, and xanthine oxidase (XO) were obtained from Sigma-Aldrich Chemical Co (St. Louis, MO, USA). All of the HPLC-grade solvents were acquired from Merck (Darmstadt, Germany).

### 2.3. Animals

Male C57BL/6 mice (20–25 g) were obtained from BioLASCO Taiwan Co., Ltd. They were housed in groups of five, which were chosen at random, in wire-mesh cages (39 cm × 26 cm × 21 cm) in a temperature (23 ± 1 °C) and humidity (60%) regulated environment with a 12 h–12 h light/dark cycle (light phase 08:00 to 20:00). The experimental protocol (CMUIACUC-103-125-N) was approved by the Institutional Animal Care and Use Committee of China Medical University. Then, the mice were cared for according to the Guiding Principles for the Care and Use of Laboratory Animals. After one week of acclimatization, all of the mice were used in an MPTP-induced PD mouse model.

### 2.4. Drug Treatment and Experimental Design

**Figure 1:**
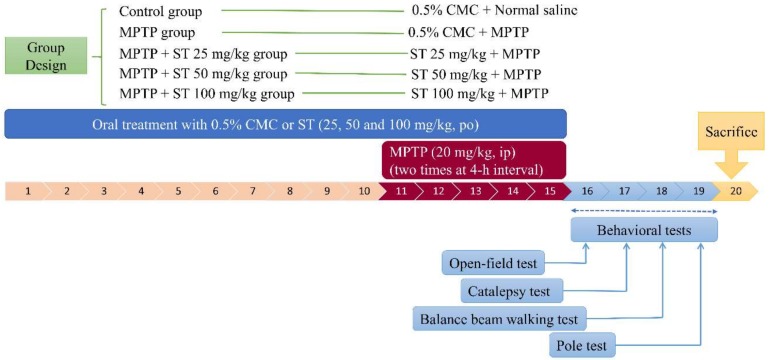
The schedule of drug treatments and behavioral tests. *Sophora tomentosa* (25 mg/kg, 50 mg/kg, and 100 mg/kg, po) was administered for 15 consecutive days until the behavioral tests. 1-methyl-4-phenyl-1,2,3,6-tetrahydropyridine (MPTP) (20 mg/kg, ip) were given two times at four-hour interval daily for five days until behavioral tests. CMC: carboxymethylcellulose, MPTP: 1-methyl-4-phenyl-1,2,3,6-tetrahydropyridine, ST: *Sophora tomentosa*. po:per os; ip:intraperitoneal.

The schedule of drug treatments and behavioral tests is shown in Figure 1. The C57BL/6 mice were randomly divided into five groups (control, MPTP, and three treatment groups) of 10 mice each. In the control group and MPTP group, mice were orally given 0.5% CMC (0.1 mL/10 g body weight) daily for 15 days until the behavioral tests. In the three treatment groups, mice were orally given ST extract (25 mg/kg, 50 mg/kg, and 100 mg/kg, respectively) daily for 15 days until the behavioral tests. All of the mice except those in the control groups were intraperitoneally given two times the amount of MPTP (dissolved with normal saline, 20 mg/kg) at four-hour intervals daily for five days until the behavioral tests, while the control group received normal saline [22]. Twenty-four hours after the last administration of MPTP or normal saline, the behavioral changes of C57BL/6 mice were observed. The behavioral tests were carried out in the following order: rearing locomotor test, catalepsy test, balance beam walking test, and pole test. The following day after the pole test, the mice were sacrificed to measure the levels of dopamine and its metabolites (DOPAC and HVA), the activities of antioxidant defense system, and the protein expression of tyrosine hydroxylase (TH), α-synuclein, and GSK-3β in the striatum.

### 2.5. Behavioral Tests

To evaluate the impairment of motor function in an MPTP-induced PD mouse model, we performed four behavioral tests, including the rearing locomotor test, catalepsy test, balance beam walking test, and pole test. Firstly, the rearing motor activities of each C57BL/6 mouse were performed with an open-field task (Coulbourn Instruments L.L.C., Holliston, MA, USA) the following day (16th day) after the last administration of 0.5% CMC or ST extract. Each C57BL/6 mouse was observed for 10 min after five min of adaption to record the rearing count and time using TruScan software v 2.07 (Coulbourn Instruments L.L.C.) [23]. The next day (17th day) after the rearing locomotor test, the catalepsy test was performed to detect muscle rigidity and failure in MPTP-induced Parkinsonism. Both forepaws of each C57BL/6 mouse were placed on a horizontal bar (0.2 cm in diameter, 65 cm in length) that was elevated 15 cm from the floor. The time for which the mouse maintained this position until it lifted its hindpaws onto the bar was recorded [24]. The next day after the catalepsy test, the balance beam walking test was performed to detect the impairment of motor coordination and balance in MPTP-induced Parkinsonism. Each C57BL/6 mouse was placed on a balance beam (1.0 cm in width, 65 cm in length) that was elevated 15 cm from the floor and tempted with fodder to cross it. The test was stopped and repeated again if the mouse fell off. The time that a mouse successfully transversed the balance beam was recorded. The test was repeated three times at five-minute intervals, and the mean value of the time was presented as the data of the balance beam walking test for analysis [25]. Finally, the pole test was performed to detect the impairment of limb movement in MPTP-induced Parkinsonism. A ball was attached to the top of a vertical pole (1.0 cm in diameter, 65 cm in length) that was wrapped with a double layer of gauze to prevent slipping. Then, each C57BL/6 mouse was placed head upward on the ball, and the time that it took for the mouse took to climb down to the floor was recorded [26].

### 2.6. Immunohistochemical Stain of TH-Positive Neurons in the SN

The day after completing the behavioral tests, all of the C57BL/6 mice were sacrificed according to ethical principles. The brains were removed carefully and fixed in 10% formalin. After post-fixation, paraffin brain slices were prepared and cut into sections (10 μm) from Bregma in the coronal section at a distance of 2.5 mm from each other and up to 3.5 mm in width using a microtome (Leica 2030 Biocut). Some sections were removed, filled with paraffin, rehydrated, and incubated with a mouse anti-tyrosine hydroxylase (TH) monoclonal antibody (Santa Cruz Biotechnology, Dallas, TX, USA). Immunolabeled sections were developed with 0.05% diaminobenzidine using a Vectastain kit (Vector Laboratories, Burlingame, CA, USA). Digital pictures were taken using 10× objectives (Nikon, Tokyo). Results were expressed as the average intensity of the TH-positive immunoreactive neurons (positive pixels) divided by the full area captured (mm^2^) using the image processing and analysis using Java software (Windows version, National Institutes of Health, Bethesda, MD, USA).

### 2.7. Measurement of Striatal Dopamine and Its Metabolite Levels

To measure the levels of striatal dopamine and its metabolites (DA and HVA), all of the C57BL/6 mice were sacrificed, and their striatal area was collected. The striatal tissues of C57BL/6 mice were homogenized in 9× ice-cold phosphate buffered saline with a glass grinder and centrifuged at 12,000 rpm for 15 min at 4 °C. Then, the aliquots of the supernatants were collected and stored at −80 °C until use. The concentrations of dopamine and its metabolites (DOPAC and HVA) of brain supernatants were measured by HPLC with electrochemical detection (EICOM HTEC-500, Kyoto, Japan) [23].

### 2.8. Biochemical Assays

The above supernatants were also used to determine the antioxidant enzyme activities for SOD, GPx, GR, and catalase, and the levels of MDA and GSH. The activities of antioxidant enzymes and the levels of MDA and GSH were measured with a spectrophotometric microplate reader (Bio-Tek, PowerWave X340, Winooski, VT, USA), according to our previous report [27]. First, SOD activity was measured kinetically with the production of nitroblue tetrazolium at 560 nm for five minutes. GPx and GR activities were measured by Cayman assay kits (Cayman Chemical Company, Ann Arbor, MI, USA). Catalase activity was determined with the decrease in the absorbance of amplex red at 560 nm. SOD and catalase activities were expressed as U/mg of protein. GPx and GR activities were expressed as mU/mg of protein. GSH levels were measured kinetically with the production of 5-thio-2-nitrobenzoic acid (TNB) from DTNB at 405 nm for five minutes. GSH levels were expressed as mmol/mg of protein. MDA levels were measured at 532 nm by the thiobarbituric acid reactive substances (TBARS) assay and expressed as MDA equivalents (mmol MDA/mg of protein).

### 2.9. Western Blotting

The striatal tissues of C57BL/6 mice were subjected to Western blot analyses to determine the protein expression of GSK-3β, *p*-GSK-3β, α-synuclein, and TH. Briefly, the striatal tissues were homogenized in 9× cold lysis buffer (20 mM of HEPES(4-(2-hydroxyethyl)-1-piperazineethanesulfonic acid) pH 7.0, 10 mM of KCl, and 0.5% NP-40) with a tissue grinder. The homogenate was incubated for 10 min and centrifuged at 12,000 rpm for 20 min to obtain the cytoplasmic supernatant. The aliquots of cytoplasmic supernatants were stored at –80 °C until use. The protein concentration was quantified using a Bradford protein assay kit (Bio-Rad Ltd. Inc., Hercules, CA, USA) and followed by electrophoretic separation through SDS-PAGE. After transferring the protein samples to PVDF(polyvinylidene difluoride) membranes, the samples were blocked with 5% non-fat dry milk and 0.1% tween-20 in tris-buffered saline at room temperature for one hour. Then, the membranes were incubated with primary antibodies against TH (Cell Signalling Technology, Danvers, MA, USA), α-synuclein (BD Biosciences, San Diego, CA, USA), phospho GSK-3β (*p*-GSK-3β), and GSK-3β (Santa Cruz Biotechnology, Dallas, TX, USA) overnight at 4 °C, and subsequently incubated with horseradish peroxidase-conjugated goat anti-rabbit or goat anti-mouse immunoglobulin G (IgG). The images were scanned using an LAS-4000 mini imaging system (Fujifilm, Kanagawa, Japan), and the optical density data was analyzed using MultiGauge v3.0 software (Fujifilm, Kanagawa, Japan). β-Actin (Proteintech, Rosemont, IL, USA) served as an internal control.

### 2.10. Determination of Radical Scavenging Activity In Vitro

The scavenging activities of ST extract against the DPPH or ABTS radicals were determined with spectrophotometric microplate readers (Bio-Tek, PowerWave X340, Winooski, VT, USA), according to the method described in our previous studies [27,28]. The scavenging activities of ST extract against the DPPH radical is expressed as gallic acid equivalents in milligram per gram of sample (GAERSC values). The scavenging activities of ST extract against the ABTS radical is expressed as trolox equivalents in mmole per gram of sample (TEAC values). Ferric reducing antioxidant power (FRAP) assay was performed according to the method described in our previous report [28]. The results are expressed as the relative ascorbic acid equivalents in mmol per gram of sample (FRAP values).

### 2.11. Determination of Antioxidant Phytoconstituent Contents by a Spectrophotometric Reader

The contents of antioxidant phytoconstituents such as total phenolics and flavonoids in the ST extract were assayed using 96-well microtiter spectrophotometric methods, according to our previous reports [27,28]. The total phenolic contents in the ST extract were measured through a redox reaction with FCP reagent and expressed as mg of gallic acid equivalents per gram of sample. The flavonoid contents in the ST extract were measured through the formation of colored products by flavonoids with aluminum salt and expressed as mg of quercetin equivalents per gram of sample.

### 2.12. Determination of Phytochemical Compounds by HPLC-DAD

ST extract was dissolved in distilled water and then filtered using an 0.22-μm filter. The stock solutions of gallic acid, protocatechuic acid, catechin, and epicatechin were prepared in methanol. All of the standard and sample solutions were injected into 20 μL in triplicate. The Shimadzu VP series HPLC and Class-VP^TM^ chromatography data system (Shimadzu Co., Kyoto, Japan) were used. A LiChrospher® RP-18e (250 × 4 mm, 5 μm) column (Merck KGaA, Darmstadt, Germany) was used. The chromatographic separation of gallic acid and gallocatechin was carried out at room temperature (RT) using a two-solvent system. Solvent A was 100% methanol, and solvent B was 0.05% phosphoric acid. The analyses were performed by a gradient program. The gradient condition was modified from the report of Fernandes et al. [29] as follows: initial with 90% solvent B, zero to 10 min maintained in 90% solvent B, 10–13 min changed to 70% solvent B, 13–18 min changed to 60% solvent B, 18–21 min changed to 40% solvent B, 21–23 min maintained in 23% solvent B, and 23–30 min changed to 90% solvent B. Signals were detected at 272 nm. The range of calibration curve was between 0.5–50 μg/mL of gallic acid, and there was a good linearity (*R^2^* = 0.9999) within this range. The range of the calibration curve was between 1–100 μg/mL of protocatechuic acid, catechin, and epicatechin, and good linearity (*R^2^* = 0.9999 for protocatechuic acid and epicatechin, and *R^2^* = 0.9997 for catechin) was also achieved within the ranges. The chromatographic peaks of all of the standards were confirmed by comparing their retention times and ultraviolet (UV) spectra.

### 2.13. Statistical Analysis

The data from behavioral tests, the concentrations of dopamine and its metabolites, the activities of the antioxidant defense system, and protein expression are presented as mean ± SEM. Data were analyzed statistically by a one-way analysis of variance (ANOVA), followed by Dunnett’s test, using statistical software SPSS 20.0 for Windows. Probability values of less than 0.05 were considered statistically significant.

## 3. Results

### 3.1. Effects of ST Extract on the Impairment of Motor Function in an MPTP-Induced PD Mouse Model

**Figure 2:**
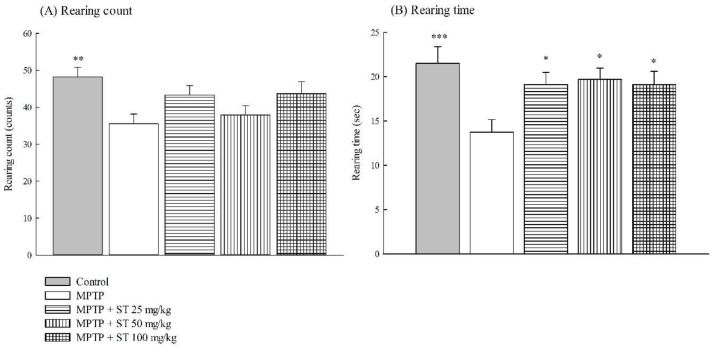
Effect of *Sophora tomentosa* extract (25 mg/kg, 50 mg/kg, and 100 mg/kg, po) on (A) rearing count and (B) rearing time on an open field test in an MPTP-induced PD mouse model. Data are expressed as mean ± SEM (*n* = 10). * *p* < 0.05, ** *p* < 0.01, *** *p* < 0.001, compared with the MPTP group. *Sophora tomentosa* was administered for 15 consecutive days until the behavioral tests. MPTP (20 mg/kg, intraperitoneal) were given two times at four-hour intervals daily for five days until behavioral tests. MPTP: 1-methyl-4-phenyl-1,2,3,6-tetrahydropyridine, PD: Parkinson’s disease, ST: *Sophora tomentosa*.

Due to the motor deficits including rigidity, bradykinesia, and postural instability being the major clinical symptoms of PD, we evaluated the effects of ST extract on the impairment of motor function with four behavioral tests, including the open field test, catalepsy test, balance beam walking test, and pole test in an MPTP-induced PD mouse model. Twenty-four hours after subacute (five-day) MPTP administration, MPTP decreased the rearing count and time in the open field test in C57BL/6 mice (*p* < 0.01, *p* < 0.001) (Figure 2). ST extract at 25 mg/kg, 50 mg/kg, and 100 mg/kg increased the rearing time, but not the count, which was decreased by MPTP in C57BL/6 mice (*p* < 0.05) (Figure 2).

**Figure 3:**
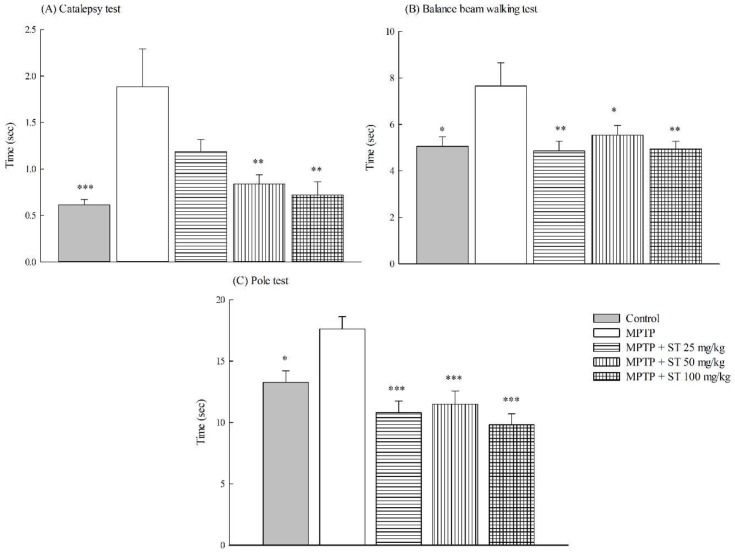
Effect of *Sophora tomentosa* extract (25 mg/kg, 50 mg/kg, and 100 mg/kg, po) on (**A**) the catalepsy test, (**B**) the balance beam walking test, and (**C**) the pole test in an MPTP-induced Parkinson disease (PD) mouse model. Data are expressed as mean ± SEM (*n* = 10). * *p* < 0.05, ** *p* < 0.01, *** *p* < 0.001, compared with the MPTP group. *Sophora tomentosa* was administered for 15 consecutive days until the behavioral tests. MPTP (20 mg/kg, ip) were given two times at four-hour intervals daily for five days until behavioral tests. MPTP: 1-methyl-4-phenyl-1,2,3,6-tetrahydropyridine, PD: Parkinson’s disease, ST: *Sophora tomentosa*.

After subacute (five-day) MPTP administration, the duration of immobility onto the bar in the catalepsy test in the MPTP group was longer than that in the control group (*p* < 0.001). ST extract at 25 mg/kg, 50 mg/kg, and 100 mg/kg decreased the duration of immobility onto the bar compared to the MPTP group in C57BL/6 mice (*p* < 0.01) (Figure 3A). In the balance beam walking test, MPTP prolonged the walking time in C57BL/6 mice (*p* < 0.05). ST extract at 25 mg/kg, 50 mg/kg, and 100 mg/kg shortened the walking time compared to the MPTP group in C57BL/6 mice (*p* < 0.05, *p* < 0.01) (Figure 3B). In the pole test, the time that it took the mice to climb from the top of the pole to the ground in the MPTP group was longer than that in the control group (*p* < 0.05). ST extract at 25 mg/kg, 50 mg/kg, and 100 mg/kg shortened the descending time compared to the MPTP group in C57BL/6 mice (*p* < 0.001) (Figure 3C).

### 3.2. Effects of ST Extract on the Nigral Dopaminergic Neurons in an MPTP-Induced PD Mouse Model

**Figure 4:**
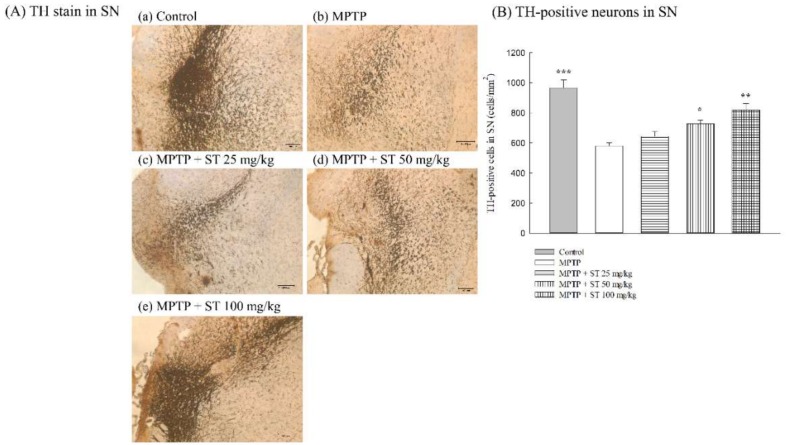
Effect of *Sophora tomentosa* extract (25 mg/kg, 50 mg/kg, and 100 mg/kg, po) on (**A**) Tyrosine hydroxylase (TH) stain and (**B**) TH-positive neurons in the substantia nigra of an MPTP-induced PD mouse model. Data are expressed as mean ± SEM (*n* = 3). * *p* < 0.05, ** *p* < 0.01, *** *p* < 0.001, compared with the MPTP group. *Sophora tomentosa* was administered for 15 consecutive days until the behavioral tests. MPTP (20 mg/kg, ip) were given two times at four-hour intervals daily for five days until the behavioral tests. MPTP: 1-methyl-4-phenyl-1,2,3,6-tetrahydropyridine, PD: Parkinson’s disease, SN: substantia nigra, ST: *Sophora tomentosa*, TH: tyrosine hydroxylase.

Due to the neuropathological role of the nigrostriatal dopaminergic pathway in PD, we assayed the effects of ST extract on the nigral dopaminergic neurons in an MPTP-induced PD mouse model by immunohistochemical staining. Five days after subacute (five-day) MPTP administration, MPTP decreased the number of TH-positive immunoreactive neurons compared to the control group in C57BL/6 mice (*p* < 0.001) (Figure 4A(a,b),B). ST extract at 50 mg/kg and 100 mg/kg restored the number of TH-positive immunoreactive neurons compared to the MPTP group in C57BL/6 mice (*p* < 0.05, *p* < 0.01) (Figure 4A(c–e),B).

### 3.3. Effects of ST Extract on the Striatal Neruochemical Alteration in an MPTP-Induced PD Mouse Model

**Figure 5:**
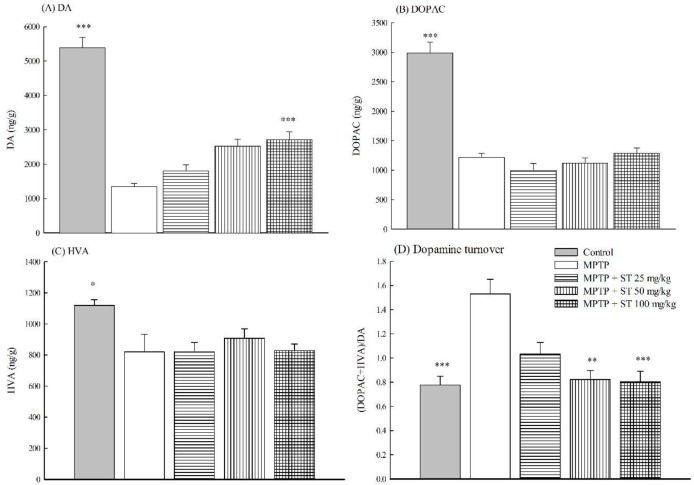
Effect of *Sophora tomentosa* extract (25 mg/kg, 50 mg/kg, and 100 mg/kg, po) on (**A**) dopamine (DA) levels, (**B**) 3,4-dihydroxyphenylacetic acid (DOPAC) levels, (**C**) homovanillic acid (HVA) levels, and (**D**) dopamine turnover in the striatum of an MPTP-induced PD mouse model. Data are expressed as mean ± SEM (*n* = 5). * *p* < 0.05, ** *p* < 0.01, *** *p* < 0.001, compared with the MPTP group. *Sophora tomentosa* was administered for 15 consecutive days until the behavioral tests. MPTP (20 mg/kg, ip) were given two times at four-hour intervals daily for five days until behavioral tests. DA: dopamine, DOPAC: 3,4-dihydroxyphenylacetic acid, HVA: homovanillic acid, MPTP: 1-methyl-4-phenyl-1,2,3,6-tetrahydropyridine, PD: Parkinson disease, ST: *Sophora tomentosa*.

Due to the role of the striatal dopaminergic dysfunction in PD, we measured the effects of ST extract on the concentrations of striatal dopamine and its metabolites (DOPAC and HVA) in an MPTP-induced PD mouse model. Five days after subacute (five-day) MPTP administration, MPTP decreased the concentrations of striatal dopamine (77.0% loss) and its metabolites (DOPAC (63.3% loss) and HVA (32.9% loss)) compared to the control group in C57BL/6 mice (*p* < 0.01, *p* < 0.001) (Figure 5A–C). ST extract only at 100 mg/kg restored the concentrations of striatal dopamine, but not its metabolites (DOPAC and HVA) compared to the MPTP group in C57BL/6 mice (*p* < 0.001) (Figure 5A–C). Furthermore, MPTP increased the striatal dopamine turnover ((DOPAC+HVA)/DA) (182%) compared to the control group in C57BL/6 mice (*p* < 0.001) (Figure 5D). ST extract at 50 mg/kg and 100 mg/kg decreased the striatal dopamine turnover, which was increased by subacute (five-day) MPTP administration (*p* < 0.01, *p* < 0.001) (Figure 5D).

### 3.4. Effects of ST Extract on the Striatal Oxidative Stress in MPTP-Induced PD Mouse Model

To clarify the role of the antioxidative defense system and oxidative damage on the effects of ST extract against MPTP-induced Parkinsonism in C57BL/6 mice, we measured the activities of the striatal antioxidant defense system, including GSH levels, the activities of antioxidant enzymes, and the levels of oxidative damage markers such as MDA in the mouse striatum. Five days after subacute (five-day) MPTP administration, MPTP decreased the activities of antioxidant enzymes including SOD, GPx, GR, and catalase in mouse striatum (*p* < 0.05) (Table 1). We further found that MPTP also decreased GSH levels, but increased MDA levels in the striatum of C57BL/6 mice (*p* < 0.05, *p* < 0.01) (Table 1). ST extract only at 100 mg/kg restored the striatal GSH levels and the activities of striatal antioxidant enzymes, including SOD, GPx, GR, and catalase, which were decreased by MPTP in C57BL/6 mice (*p* < 0.05, *p* < 0.001) (Table 1). ST extract only at 100 mg/kg decreased striatal MDA levels, which was elevated by subacute (five-day) MPTP administration (*p* < 0.01) (Table 1).

### 3.5. Effects of ST Extract on the Striatal Protein Expression in MPTP-Induced PD Mouse Model

**Figure 6:**
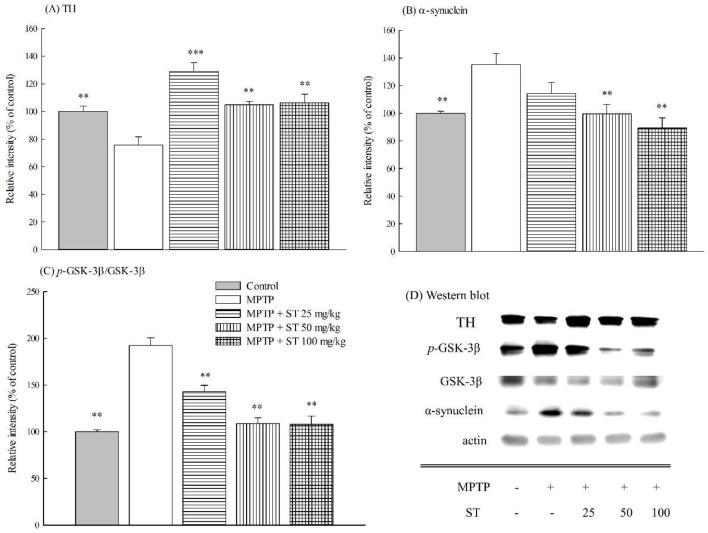
The effect of *Sophora tomentosa* extract (25 mg/kg, 50 mg/kg, and 100 mg/kg, po) on (**A**) the expression of tyrosine hydroxylase, (**B**) the expression of α-synuclein expression, (**C**) the ratio of *p*-GSK-3β/GSK-3β, and (**D**) protein was determined by immunoblot assay in an MPTP-induced PD mouse model. Data are expressed as mean ± SEM (*n* = 5). ** *p* < 0.01, *** *p* < 0.001, compared with the MPTP group. *Sophora tomentosa* was administered for 15 consecutive days until the behavioral tests. MPTP (20 mg/kg, ip) were given two times at four-hour daily intervals for five days until behavioral tests. GSK-3β: glycogen synthase kinase 3β, MPTP: 1-methyl-4-phenyl-1,2,3,6-tetrahydropyridine, PD: Parkinson disease, *p*-GSK-3β: phospho GSK-3β, ST: *Sophora tomentosa*, TH: tyrosine hydroxylase.

Immunoblotting was used to determine the effect of ST extract on the protein expression of TH, α-synuclein, *p*-GSK-3β, and GSK-3β in the striatum of an MPTP-induced PD mouse model. The protein immunoblot assay is shown in Figure 6D. Five days after subacute (five-day) MPTP administration, MPTP decreased the expression of TH protein (*p* < 0.01) (Figure 6A), but increased the expression of α-synuclein protein in mouse striatum (*p* < 0.01) (Figure 6B). Furthermore, MPTP increased the ratio of *p*-GSK-3β protein versus GSK-3β protein in mouse striatum (*p* < 0.01) (Figure 6C). ST extract at 25–100 mg/kg restored the expression of striatal TH protein (*p* < 0.01, *p* < 0.001) and inhibited the elevated ratio of *p*-GSK-3β protein versus GSK-3β protein in mouse striatum compared to the MPTP group (*p* < 0.01) (Figure 6A,C). However, ST extract only at 50–100 mg/kg inhibited the elevated expression of striatal α-synuclein protein that was caused by the subacute (five-day) MPTP administration (*p* < 0.01) (Figure 6B).

### 3.6. Radical Scavenging Capacities

The 50% scavenging capacity (IC_50_) of gallic acid against a DPPH radical with a 96-well microtiter spectrophotometric method is 1.68 ± 0.05 μg/mL. The GAERSC value of ST extract against DPPH radical is 3.33 ± 0.20 mg gallic acid/g ST extract. The 50% scavenging capacity (IC_50_) of trolox against the ABTS radical with a 96-well microtiter spectrophotometric method is 12.47 ± 0.05 μM. The TEAC value of ST extract against the ABTS radical is 62.27 ± 0.64 μmol trolox/g ST extract. The FRAP value of ST extract is 78.16 ± 0.38 mg ascorbic acid/g ST extract.

### 3.7. Antioxidant Phytoconstituents Contents

The present study also assayed the contents of the antioxidant phytoconstituents, including total phenolics and flavonoids in ST extract, using some 96-well microtiter spectrophotometric methods. The total phenolic content in each gram of ST extract is equal to 61.21 ± 0.08 mg of gallic acid. The total flavonoid content in each gram of ST extract is equal to 3.70 ± 0.12 mg of quercetin.

### 3.8. Phytochemical Profiles of ST Extract by HPLC-DAD

**Figure 7:**
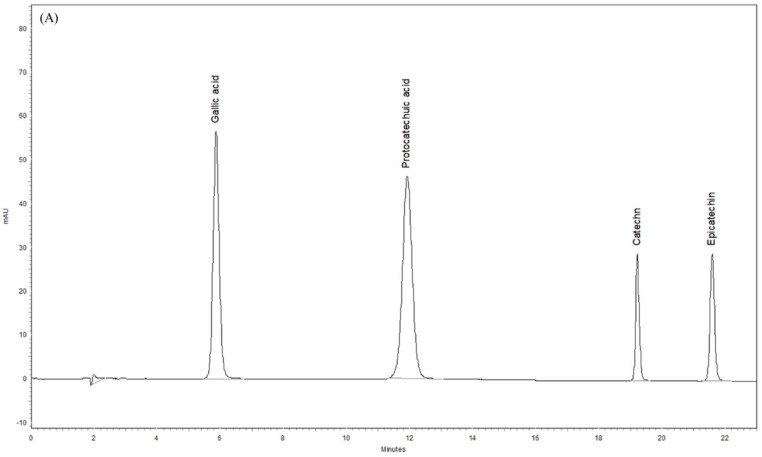

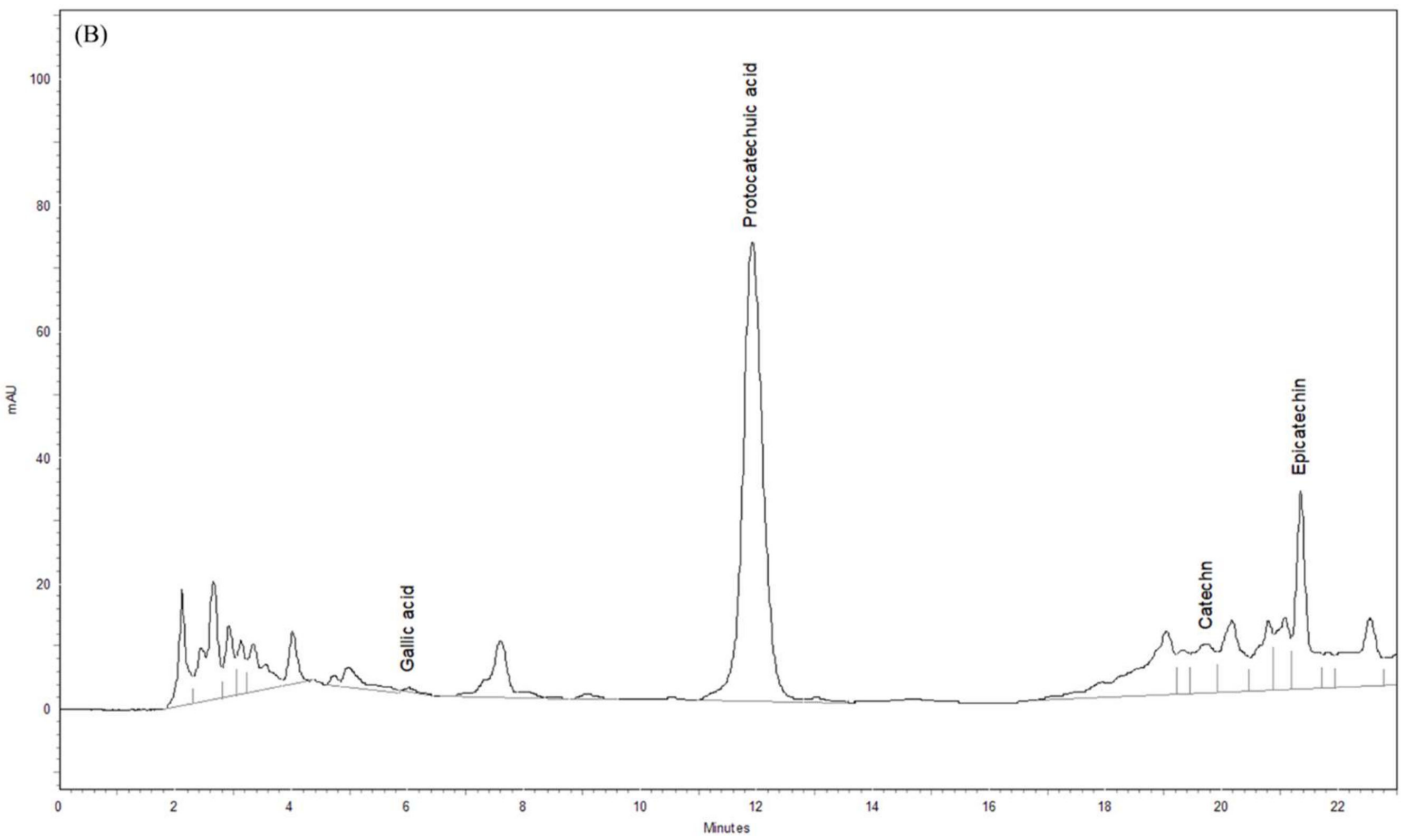
High-performance liquid chromatography (HPLC) chromatograms of (**A**) standards and (**B**) *Sophora tomentosa* extract at 272 nm.

The phytochemical profile of ST extract was further assayed using HPLC-DAD. The chromatographs of standards and ST extract are shown in Figure 7A,B. There are some major peaks in the chromatograph of ST extract, especial protocatechuic acid (11.97 min) and epicatechin (21.96 min). Compared with the retention times and UV spectra of the standard chromatograph, each gram of ST extract contained 12.22 ± 0.07 mg of protocatechuic acid, 6.51 ± 0.24 mg of epicatechin, 3.77 ± 0.11 mg of catechin, and 23.59 ± 2.81 μg of gallic acid.

## 4. Discussion

The major clinical symptoms of PD are the behavioral alterations including tremor, rigidity, bradykinesia, and postural instability. After acute, subacute, or chronic administration, the systemic injection of MPTP in C57BL/6 mice replicated almost all of the pathological hallmarks of human PD patients. Sophora species, especially SF and SJ, have been used to prevent aging-associated neurodegenerative disorders such as PD or ischemia/reperfusion in traditional Chinese medicine. Kim et al. indicated that SF extract protected against MPP^+^-induced neurotoxicology in SH-SY5Y neuroblastoma cells [17]. ST had similar therapeutic applications to these above Sophora species, but there is no related literature. Hence, the present study first evaluated the neuroprotective effects of ST extract against MPTP-induced Parkinsonism in C57BL/6 mice. The present data showed that subacute systemic injection with MPTP caused the symptom of bradykinesia in the open field test, the symptom of rigidity in the catalepsy test, and the symptom of postural instability and motor coordination in the balance beam walking test and pole test in C57BL/6 mice, in which the above motor dysfunction was same as that described by the other reports [23,24,25,26]. ST extract alleviated the motor dysfunction, including the rigidity, bradykinesia, and postural instability induced by subacute administration with MPTP. Therefore, we suggested that ST extract prevented the motor symptoms of MPTP-induced Parkinsonism in C57BL/6 mice.

The appearance of these above motor symptoms in PD is the result of the selective progressive degeneration of the dopaminergic neurons in the SN and the depletion of projecting dopaminergic nerve fibers in the striatum up to a threshold level. According to clinical reports, motor symptoms first appear in PD patients at a loss of 50–60% of dopaminergic neurons in the SN and 70–80% depletion of dopamine levels in the striatum [1,30]. MPTP is converted to MPP^+^ via monoamine oxidase B (MAO-B) in the glia when it crosses the blood–brain barrier. MPP^+^ is selectively transported by the dopamine transporter (DAT) into dopaminergic neurons in the SN rather than those in the ventral tegmental area because there is a higher density of DAT in the midbrain, which is then damaged by the dopaminergic neurons in the SN. Hence, the administration with MPTP mainly caused a decrease in the density of TH in the nigrostriatal pathway and led to a loss of projecting striatal dopaminergic nerve terminal markers—dopamine and its metabolites—in C57BL/6 mice [14,30]. Our present results found that subacute administration with MPTP decreased the density of TH-positive dopaminergic neurons in SN and the expression of TH in the striatum compared to normal mice. There is a significant change in striatal DA (77% loss) and DOPAC (63% loss) concentrations after subacute (five-day) MPTP administration compared to normal mice. These decreased activities in the nigrostriatal dopaminergic pathway were consistent with those described by other reports [14,30]. The dopamine turnover was increased by about 1.8 times after subacute (five-day) MPTP administration compared to normal mice. This reduced ratio in the concentrations of striatal dopamine and its major metabolite DOPAC caused by subacute (five-day) MPTP administration is similar to that described in the early report [22], and is consistent with the association of motor symptoms and biochemical changes in clinical reports [30]. ST extract (50 mg/kg and 100 mg/kg) restored the density of TH-positive dopaminergic neurons in SN and the expression of TH protein in the striatum decreased by subacute (five-day) MPTP administration. We further found that ST extract (100 mg/kg) reversed striatal DA concentration to about 52% and the dopamine turnover to only about 0.48 times compared to normal mice. Hence, the increase of TH-positive dopaminergic neurons in SN and the upregulation of TH expression in the striatum corroborates to an increase in dopamine levels and a decrease in dopamine turnover in mouse striatum with an ST extract treatment. The increase in striatal dopamine levels also corroborates to the alleviation of motor functions with ST extract treatment in an MPTP-induced PD mouse model.

Oxidative stress, a major biochemical pathogenic factor of PD, is the imbalance between the levels of intracellular free radicals and the activities of the intracellular antioxidant defense system [1,2,4]. Excess intracellular free radicals attacked the adjacent biomolecules such as polyunsaturated fatty acids, proteins, and nuclei acids, and then caused lower activities of the intracellular antioxidant defense systems such as SOD, catalase, and GSH recycling, including GSH and related enzymes, such as GPx and GR. Once MPP^+^ are released into the dopaminergic cells, MPP^+^ can enter into mitochondria, where it interferes with complex I of the electron transport chain. This blockade caused the generation of the oxygen free radicals that rearranges to form hydrogen peroxide and leads to the formation of hydroxyl radicals. So, MPTP can lead to some biochemical pathogenesis, such as oxidative damage and the dysfunction of nigrostriatal dopaminergic system such as PD [3,14,30]. Our results are consistent with earlier reports [31,32] that subacute (five-day) MPTP administration decreased the levels of GSH and the activities of antioxidant enzymes, and increased the levels of oxidative damage marker MDA in C57BL/6 mice striatum. We found that ST extract (100 mg/kg) restored the levels of GSH and the activities of antioxidant enzymes that were decreased by subacute (five-day) MPTP administration, thereby decreasing the oxidative damage in C57BL/6 mice. Therefore, we suggest that ST extract prevented motor deficits and dopaminergic dysfunction in an MPTP-induced PD mouse model partially via upregulating the activities of the striatal antioxidant status.

The abnormal processing of cellular proteins is another major biochemical pathogenic factor of PD. α-Synuclein is a major component of cellular protein that localizes to the presynaptic terminal in normal physiological conditions and regulates synaptic functions, including synaptic–vesicle trafficking and fusion. Some evidences support the notion that increased oxidative stress in the brain may contribute to α-synuclein aggregation, overexpression, or misfolding. α-Synuclein accumulates in neuronal cell bodies and processes to form Lewy bodies and Lewy neurites in the PD brain. The mutation or overexpression of α-synuclein also caused the degeneration of dopaminergic neurons [6,7]. The overexpression of α-synuclein in SH-SY5Y neuroblastoma cells led to elevated cellular mortality and synaptic degeneration via the decrease in antioxidant defense [8,9,10]. The hypothesis about α-synuclein pathology claimed that the biology of α-synuclein is altered by oxidative stress, while α-synuclein aggregation, overexpression, or misfolding have greater neurotoxicity and create a feed-forward state of progressive dopaminergic neuronal death in PD patients. A single injection of MPTP in primates caused α-synuclein pathology [33]. Recent reports indicated that systemic injection with MPTP in C57BL/6 mice induced α-synuclein overexpression and aggregation [34,35]. Our present results found that subacute (five-day) MPTP administration increased the expression of α-synuclein in C57BL/6 mice striatum. ST extract prevented the expression of striatal α-synuclein, which was elevated by subacute (five-day) MPTP administration. Furthermore, accumulating evidences have revealed that GSK-3β and α-synuclein have a mutual interaction with each other. In the postmortem striatum of PD patients, α-synuclein accumulation and the active form of GSK-3β are localized in the same areas [11]. In α-synuclein transgenic mice, α-synuclein overexpression was also associated with the elevation of active GSK-3β [10]. GSK-3β inhibitors abrogated the impairment of motor function, α-synuclein accumulation, and neurotoxicity that was induced by MPP^+^ [12]. On the other hand, GSK-3β is known to play critical roles in oxidative stress-induced neuronal apoptosis and the pathogenesis of PD [36]. Hence, GSK-3β inhibitors were suggested as a potential therapeutic strategy in PD. Our present results found that subacute (five-day) MPTP administration increased GSK-3β phosphorylation in C57BL/6 mice striatum. ST extract prevented striatal GSK-3β phosphorylation, which was elevated by subacute (five-day) MPTP administration. Hence, we suggest that ST extract prevented the motor deficits in an MPTP-induced PD mouse model partially through upregulating the activities of striatal antioxidant status and downregulating the phosphorylation of GSK-3β to decrease α-synuclein overexpression and nigrostriatal dopaminergic dysfunction.

The antioxidant activities of a plant are closely correlated to the contents of its phenolic compounds such as total phenolics and flavonoids. Sophora species such as SF and SJ extracts exert the antioxidant and neuroprotective potency mainly via their flavonoid phytoconstituents [15,18,20]. Our present data demonstrated that ST extract had the higher total phenolic contents (61.21 mg gallic acid/g ST extract) and better ABTS radical scavenging potency. We further found out that ST extract contained some phenolic compounds, including gallic acid, protocatechuic acid, catechin, and epicatechin, and there are higher contents of protocatechuic acid (12.22 mg/g of ST extract) and epicatechin (6.51 mg/g of ST extract) in ST extract. Many researchers have indicated that protocatechuic acid and epicatechin have the therapeutic potential in neurodegenerative diseases [37,38]. Protocatechuic acid protected neurotoxicity and apoptosis induced by 6-hydroxydopamine (6-OHDA), MPTP, rotenone, and hydrogen peroxide via restoring intracellular antioxidant activities and ameliorating mitochondria function in PC12 cells [39,40,41,42]. These neuroprotective effects of protocatechuic acid might be also believed to inhibit the abnormal oligomerization and aggregation of α-synuclein [40,43]. Furthermore, protocatechuic acid (50–100 mg/kg) has neuroprotective effects against MPTP-induced damage in C57BL/6J mice by increasing the contents of dopamine and its metabolites in striatum, and by upregulating the expression of TH in SN [44]. Epicatechin protected neurotoxicity induced by methamphetamine via reducing ER stress and mitochondrial damage in HT22 hippocampal neuronal cells [45]. Also, epicatechin (10–100 mg/kg) is effective against MPP(+)-induced and 6-OHDA-induced biochemical and behavioral damage via restoring intracellular antioxidant activities in rats [46,47]. The neuroprotective effects of ST extract are consistent with those of the above two phytoconstituents, although the contents of protocatechuic acid and epicatechin in ST extract is lower than the used dose that has been described in other reports [44,46,47]. Hence, we suggested that protocatechuic acid and epicatechin are major active compounds in ST extract against an MPTP-induced PD mouse model. This neuroprotective mechanism of ST extract against MPTP-induced Parkinsonism, which was the same as protocatechuic acid, might be related to decreasing the phosphorylation of GSK-3β and restoring the activities of striatal antioxidant defenses to restore the nigrostriatal dopaminergic function and decrease α-synuclein accumulation. Further investigation on the synergistic effects of protocatechuic acid and epicatechin and the further isolation and activity assay of other active phytoconstituents in ST extract are still necessary. Moreover, most of the in vitro research reports about the neuroprotective effects of protocatechuic acid and epicatechin have indicated that these two compounds exerted the neuroprotective effects through reducing endoplasmic reticulum (ER) stress and mitochondrial damage [41,45]. Therefore, the role of the signaling pathways from the ER and mitochondria in the neuroprotective effects of ST extract and its active phytoconstituents against MPTP-induced Parkinsonism must be further investigated.

## 5. Conclusions

**Figure 8:**
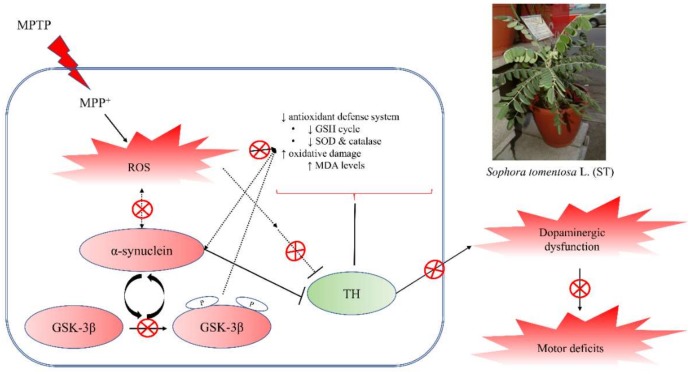
The biological action of *Sophora tomentosa* (ST) as a potential neuroprotective plant against an MPTP-induced PD mouse model. GSH: glutathione, MDA: malondialdehyde, MPTP: 1-methyl-4-phenyl-1,2,3,6-tetrahydropyridine, PD: Parkinson’s disease, ROS: reactive oxygen species, SOD: superoxide dismutase, ST: *Sophora tomentosa*, TH: tyrosine hydroxylase.

In conclusion, ST extract alleviated the impairment of motor function in four behavioral tests in an MPTP-induced PD mouse model. ST extract decreased the damage of TH-positive dopaminergic neurons in SN, and then restored the levels of dopamine and TH protein in the striatum. ST extract further decreased α-synuclein overexpression and GSK-3β phosphorylation in mouse striatum. Also, ST extract restored the levels of GSH and the activities of antioxidant enzymes, and decreased the elevated levels of MDA in mouse striatum. ST extract exerted higher ABTS radical scavenging capacities through its higher phenolic contents. Protocatechuic acid and epicatechin are its major active phenolic compounds, because protocatechuic acid and epicatechin have antioxidant and neuroprotective activities against the oxidative stress and neuronal damage caused by 6-OHDA, MPTP, rotenone, and hydrogen peroxide [39,40,41,42,46,47]. Hence, we suggested that ST extract has the potential to counteract MPTP-induced motor deficit and nigrostriatal dopaminergic degeneration via the regulation of GSK-3β phosphorylation, α-synuclein pathology, and oxidative stress. This neuroprotective mechanism of ST extract against MPTP-induced Parkinsonism might be related to decreasing the phosphorylation of GSK-3β and restoring the activities of striatal antioxidant defenses to restore the nigrostriatal dopaminergic function and decrease α-synuclein accumulation (Figure 8).

## Figures and Tables

**Table 1:** Effects of *Sophora tomentosa* (25 mg/kg, 50 mg/kg, and 100 mg/kg, po) extract on the levels of glutathione and malondialdehyde, and the activities of antioxidant enzymes in the striatum of 1-methyl-4-phenyl-1,2,3,6-tetrahydropyridine (MPTP)-induced Parkinson disease (PD) mouse model.

Samples	GSH (nmoL/mg of protein)	GR (mU/mg of protein)	GPx (mU/mg of protein)	SOD (U/mg of protein)	Catalase (U/mg of protein)	MDA (nmoL/mg of protein)
Control	2.54 ± 0.35 *	210.08 ± 12.44 *	4.86 ± 0.43 *	21.90 ± 2.71 *	11.11 ± 1.47 *	4.28 ± 0.23 **
MPTP	1.55 ± 0.25	144.02 ± 9.05	3.30 ± 0.23	12.16 ± 1.35	5.79 ± 0.82	8.11 ± 0.76
MPTP + ST 25 mg/kg	1.82 ± 0.22	145.75 ± 14.98	3.89 ± 0.27	14.48 ± 2.00	5.52 ± 1.02	8.25 ± 0.65
MPTP + ST 50 mg/kg	2.06 ± 0.22	180.04 ± 18.24	4.47 ± 0.54	18.10 ± 2.93	8.69 ± 1.93	6.43 ± 1.12
MPTP + ST 100 mg/kg	2.65 ± 0.14 *	249.23 ± 19.10 ***	5.08 ± 0.47 *	25.69 ± 2.12 ***	11.79 ± 1.17 *	4.52 ± 0.47 **

Data are expressed as mean ± SEM (*n* = 5). * *p* < 0.05, ** *p* < 0.01, *** *p* < 0.001, compared with MPTP group. *Sophora tomentosa* was administered for 15 consecutive days until the behavioral tests. MPTP (20 mg/kg, ip) were given two times at 4-h interval daily for 5 days until behavioral tests. GPx: glutathione peroxidase, GR: glutathione reductase, GSH: glutathione, MDA: malondialdehyde, MPTP: 1-methyl-4-phenyl-1,2,3,6-tetrahydropyridine, PD: Parkinson disease, SOD: superoxide dismutase, ST: *Sophora tomentosa*.
